# Effect of topical nepafenac in prevention of macular edema after cataract surgery in patients with non-proliferative diabetic retinopathy

**DOI:** 10.12669/pjms.331.11644

**Published:** 2017

**Authors:** Muhammad Haroon Sarfraz, Rana Intisar Ul Haq, Mohammad Asim Mehboob

**Affiliations:** 1 Dr. Muhammad Haroon Sarfraz, MBBS. Armed Forces Institute of Ophthalmology, Rawalpindi, Pakistan; 2 Dr. Rana Intisar Ul Haq, MCPS(Ophth), FCPS(Ophth). Armed Forces Institute of Ophthalmology, Rawalpindi, Pakistan; 3 Dr. Mohammad Asim Mehboob, MBBS. PNS Shifa Naval Hospital, Karachi, Pakistan

**Keywords:** Cataract Surgery, Macular Edema, Nepafenac, Non-Proliferative Diabetic Retinopathy

## Abstract

**Objective:**

To determine the efficacy of topical Nepafenac (0.1%), administered post-operatively in prevention of Macular Edema (ME), after cataract surgery in patients with Non-Proliferative Diabetic Retinopathy (NPDR).

**Methods:**

This randomized control trial was conducted at Armed Forces Institute of Ophthalmology (AFIO), Rawalpindi from Sep 2015 to Sep 2016. Sixty eyes of 60 patients with NPDR underwent phacoemulsification with intraocular lens implantation. Group 1 received 0.1% Nepafenac, 8-hourly, in operated eye after cataract surgery for three months, along with routine post-operative medications. Group-2 received only routine post-operative medications. ME was defined as increase in Central Macular Thickness (CMT) of >10% from pre-operative baseline, measured using spectral domain optical coherence tomography.

**Results:**

Mean age of study population was 60.97±4.91 years. Out of 60 patients, 34 (56.7%) were males and 24 (43.3%) were females. Mean pre-operative CMT, 3 months post-operative CMT, mean change in CMT and mean frequency change in CMT of Group-1 was 226.5±10.86µm, 228.83±14.56 µm, 2.33±10.45 µm and 1.05% respectively. Mean pre-operative CMT, three months post-operative CMT, mean change in CMT and mean frequency change in CMT in Group-2 was 223.93±11.69µm, 236.17±16.16 µm, 12.23±12.40µm and 5.51% respectively. ME was observed in one patient (3.3%) in Group-1, and seven patients (23.3%) in Group 2. The difference of mean change in CMT and frequency change in CMT between groups was statistically significant (p<0.05).

**Conclusion:**

0.1% topical Nepafenac is effective in prevention of macular edema after cataract surgery in patients with non-proliferative diabetic retinopathy (NPDR).

## INTRODUCTION

Diabetes Mellitus (DM) has potentially blinding effects on human eye. There is strong evidence of role of diabetes in pathogenesis of age-related cataracts. Effect of polyol pathway and induction of glycation in lens proteins following increased glucose levels in the aqueous humor have been implicated for cataract formation.^1^ Cataract surgery is therefore mandatory requirement of all diabetic patients for improving vision and vision related quality of life.

Despite advances in technique and surgical materials, cataract surgery is frequently accompanied with complications in diabetic patients. Macular Edema (ME) is the most frequent cause of reduced vision following uneventful cataract surgery, with incidence as high as 18%.^2^ With advent of high resolution Spectral Domain Optical Coherence Tomography (SD-OCT), it is possible to objectively measure ME as increase in Central Macular Thickness (CMT). ME after cataract surgery further jeopardizes vision, already worsened by Non-Proliferative Diabetic Retinopathy (NPDR). One study found an increase in CMT of at least 11% in 25.7% of the eyes with NPDR.^3^ ME is mainly caused by the accumulation of extracellular fluid within the retina due to leakage from dilated capillaries. Role of prostaglandins in the vitreous cavity as a result of rupture of inner blood retinal barrier resulting in ME has also been mentioned.^4^

Non-Steroidal Anti-Inflammatory Drugs (NSAIDS) cause Cyclo–Oxygenase enzyme inhibition and thereby inhibit the production of prostaglandins.^5^ Nepafenac is a prodrug, which is hydrolysed in the intraocular tissues to Amfenac. This active substance has good efficacy, excellent bioavailability and minimum toxic effects on ocular tissues.^6^ Inhibition of prostaglandins synthesis by Nepafenac prevents development of ME.^7^

Pakistan has a large population of diabetic patients undergoing cataract surgery. Once ME develops, the prolonged and expensive treatment is beyond affordability of majority of Pakistani population. Prevention of development of ME after cataract surgery is thus a cost effective and logical way forward.

Our objective was to determine the efficacy of topical Nepafenac (0.1%), administered post-operatively in prevention of Macular Edema (ME), after cataract surgery in patients with Non-Proliferative Diabetic Retinopathy (NPDR).

## METHODS

After approval by the hospital ethical review committee, informed written consent was taken from the patients prior to inclusion in the study. Keeping significance level of 5% and power of test at 80%, a sample size of 60 eyes was calculated.^8^ Patients from either gender, aged between 40-60 years, with non-centre involving NPDR, controlled fasting blood sugar, normal levels of HbA1C and significant cataract advocating need for cataract surgery were included. Patients with known history of ME, high myopia, ocular surgery, retinal laser photocoagulation, intravitreal injections, uveitis, glaucoma, prostaglandins use, complicated cataract surgery due to posterior capsular rupture or vitreous loss were excluded. Subjects fulfilling the inclusion criteria underwent ophthalmic examination including uncorrected and best corrected visual acuity measurement, slit lamp examination for evaluating type of cataract and confirmation of non-centre involving NPDR. Examination of all patients was performed by single researcher to exclude observer bias. On confirmation of controlled blood sugar levels, patients underwent phacoemulsification cataract surgery, under topical anesthesia, (0.5% Proparacaine Hydrochloride - Alcaine, Alcon, Switzerland) with implantation of intraocular lens by single surgeon to exclude bias. Post operatively, the eyes were randomly divided in two groups by lottery method. Group-1 was the Nepafenac group, prescribed with topical Nepafenac 0.1%, 8 hourly, started on first post-op day, for three months. Group-2 was control group.

All patients received standard post-operative topical medication (Moxifloxacin, 2%, four times daily and Prednisolone, 0.1%, 4 hourly) for two weeks. The patients underwent routine follow up at one week, two weeks, one month, two months and three months post operatively and were subjected to visual acuity measurement and measurement of CMT by SD-OCT (3D OCT-1000 Markll, Topcon Co, Tokyo, Japan). Those eyes requiring additional treatment due to post-operative pain or inflammation were excluded from the study. Efficacy was defined as increase in CMT of <10% from pre-operative value. The pre devised proforma was completed endorsing subject’s demography, ocular examination and investigations findings.

### Statistical Analysis

Data was evaluated and analyzed using Statistical Program for Social Sciences (SPSS) version 17. Mean and standard deviation was reported for continuous variables (Age, CMT) while frequency and percentage for nominal data (gender, laterality of eyes, efficacy). Shapiro Wilk’s test was used to check normality of data. Post normality testing, independent ‘t’ test was used to compare quantitative variables, and Chi Square test was used to compare qualitative variables between two groups. Paired ‘t’ test was used to compare post-operative values from pre-operative values within each group. A p value of ≤0.05 was considered statistically significant.

## RESULTS

A total of 74 patients were initially recruited. 8 patients with complicated cataract surgery and 6 failing follow up were excluded. A total of 60 eyes of 60 patients completing the follow up were thus included and analysed. Demographic details, mean pre-operative CMT, one month, two months, three months post-operative CMT, mean change in CMT, mean frequency change in CMT of study population and both groups is given in Table-I. Comparison of post-operative CMT from pre-operative value within each group is given in Table-II. ME (or no efficacy) was defined as increase in CMT of >10% from pre-operative value after 3 months.

**Table-I:** Demography and Clinical Data of Study Population (n=60).

Variable	Study Population (n=60)	Group 1 – Nepafenac Group (n=30)	Group 2 – Control Group (n=30)	p Value (Between Groups)
Age (Years) Mean ± SD	60.97±4.91	61.3±4.89	60.63±4.99	0.603
Gender (Male/Female)	34 /26 (56.7%)/ (43.3%)	15 /15 (50%)/ (50%)	19 /11 (63.3%)/ (36.7%)	0.297*
Laterality Right/Left	31 / 29 (51.7%)/(48.3%)	17/ 13 (56.7%)/(43.3%)	14 / 16 (46.7%)/(53.3%)	0.438*
Pre-Operative CMT (µm) Mean ± SD	225.22±11.26	226.5±10.86	223.93±11.69	0.382
1 month Post-Operative CMT(µm) Mean ± SD	225.87±9.55	223.43±8.62	228.30±9.96	0.048
2 months Post-Operative CMT(µm) Mean ± SD	226.70±9.97	220.87±7.28	232.53±8.90	<0.001
3 months Post-Operative CMT(µm) Mean ± SD	232.50±15.69	228.83±14.56	236.17±16.16	0.07
Mean change in CMT (µm) Mean ± SD	7.28±12.41	2.33±10.45	12.23±12.40	<0.001
Mean frequency increase in CMT (%)	3.28%	1.05%	5.51%	<0.001
Efficacy (Yes/No)	52/8 (86.7%)/ (13.3%)	29 /1 (96.7%)/ (3.3%)	23/7 (76.7%)/ (23.3%)	0.023*

* Chi Square test.

**Table-II:** Comparison of Post-operative CMT from Pre-operative Value within each Group.

Variable	Group 1 Nepafenac Group (n=30)	Group 2 Control Group (n=30)
***1 month Folllow up***		
Pre-operative CMT (µm)	226.5±10.86	223.93±11.69
1 month post-operative CMT (µm)	223.43±8.62	228.30±9.96
p Value*	<0.001	<0.001
***2 months Folllow up***		
Pre-operative CMT (µm)	226.5±10.86	223.93±11.69
2 months post-operative CMT (µm)	220.87±7.28	232.53±8.90
p Value*	<0.001	<0.001
***3 months Folllow up***		
Pre-operative CMT (µm)	226.5±10.86	223.93±11.69
3 months post-operative CMT (µm)*	228.83±14.56	236.17±16.16
p Value*	0.231	<0.001

* Paired ‘t’ Test.

## DISCUSSION

Increase in CMT is direct evidence of clinical and sub-clinical ME. Pasova P et al found increase in CMT to be transient in nature, with return to baseline after 6 months.^9^ Frequency of development of ME after cataract surgery is variable in literature. One study evaluating 15 randomized control trials suggested frequency of ME to be approximately 25%, in patients without any risk factors, treated with topical steroids only.^4^ Other studies showed development of ME even after un-complicated cataract surgery and implantation of intraocular lens, in 4 weeks follow up period.^10^

Largest analysis evaluating 81984 eyes for assessment of risk factors showed development of ME in only 1.17% of eyes without any pre-operative risk factor. Being diabetic increased the risk to 1.8 times, and having Diabetic Retinopathy (DR) increased the risk to 6.23 times. A greater chance of ME development was seen with increasing severity of DR.^11^ Kwon SI et al showed that 18% of diabetic patients developed transient ME, with 68% showing resolution after 6 months of surgery.^2^

Brito PN et al found 11% increase in CMT in 25.7% of patients with DR, not given intravitreal injection during cataract surgery.^3^ Chen XY et al in their study on 92 eyes found that patients already suffering from DR have more chances of development of ME after cataract surgery.^12^ Gallego-Pinazo R et al showed that patients with NPDR benefit from per-operative injection of Bevacizumab evidenced by less post-operative increase in CMT.^13^ Other studies have shown marked difference in CMT between normal patients and patients with NPDR undergoing cataract surgery.^14^-^16^ Giocanti-Aurégan A et al showed that change in CMT after 6 months of cataract surgery in diabetic patients without NPDR was equal to non-diabetic patients.^17^ This suggests that NPDR is a more important risk factor for development of ME after cataract surgery, than DM itself. Diabetic Retinopathy Clinical Research Network findings are the most widely stated and followed recommendations in ophthalmic literature. The multicentre trial highlighted that patient with no ME before cataract surgery did not develop ME post-operatively. Of patients with non-center involving ME pre-operatively, 10% developed centre-involving ME post-operatively.^18^ It is thus assumed that development of ME after cataract surgery, depends on multiple factors including duration, severity and control of DM, along with presence of other risk factors like hypertension, pre-existing ME, prior treatment with lasers or intravitreal injections, DR, operative complications and accuracy of SD-OCT.

Options for prevention of ME after cataract surgery in patients with NPDR include pre-operative treatment with steroids, intravitreal injections of anti-vascular endothelial growth factors, laser treatment, and topical NSAIDS. After approval in 2005, topical Nepafenac is widely prescribed to manage pain, inflammation and ME after cataract surgery. We found significant difference of CMT from baseline at one and two months in Nepafenac group, but difference was not statistically significant at three months. Control group kept on showing significant difference from baseline till 3months follow up period.

A multicentre randomized control trial comparing role of Nepafenac in prevention of ME after cataract surgery showed that ME developed in 3.2% of patients given Nepafenac, compared to 16.7% patients in vehicle group.^8^ However, Cervantes-Coste G et al found no efficacy of Nepafenac, as compared to control group in terms of change in CMT, though the follow up period was only 6 weeks.^19^

Varying results are found in literature comparing efficacy of different NSAIDS for prevention of ME. Studies measuring prostaglandins in aqueous show minimum levels of prostaglandins in patients given pre-operatively Nepafenac as compared to Ketorolac and Bromefenac.^7^ However, other studies comparing Nepafenac with Ketorolac in terms of pain and discomfort have shown equal efficacy of these drugs.^20^ Another study found increased concentrations of prostaglandins after pre-operative use of Nepafenac, as compared to Ketorolac and Bromefenac.^21^ We did not find any study comparing effects of Nepafenac in terms of change in CMT from baseline, with other NSAIDS. Since Nepafenac is clinically useful for management of post-operative pain, continuous topical use can provide added advantage of preventing ME in high risk patients with NPDR.

We used 1% Prednisolone Acetate topically for management of post-operative inflammation in all patients for two weeks. As a standard protocol followed after cataract surgery, Prednisolone along with Moxifloxacin has proven to be effective in managing post-operative inflammation and wound care. Since ME developed in seven patients in control group, as compared to one in Nepefenac group, we find short term use of Prednisolone to be in-effective in prevention of ME after cataract surgery. Studies comparing combination of Nepafenac and Dexamethasone with Dexamethasone alone in prevention of ME and change in macular volume after cataract surgery have shown clear benefit of use of combination.^5^ Another study comparing combination of Ketorolac and Dexamethasone with Dexamethasone alone have shown four times more patients developing ME in Dexamethasone alone group.^22^ Therefore, there is sufficient evidence advocating use of topical nepafenac in adjunction with topical steroids for prevention of ME in patients with NPDR.

## CONCLUSION

Nepafenac has advantage in prevention of ME after cataract surgery in patients with NPDR. We recommend its use for at least three months after cataract surgery, with continuous follow up in high risk patients with NPDR.
